# Modeling photoacoustic pressure generation in colloidal suspensions at different volume fractions based on a multi-scale approach

**DOI:** 10.1016/j.pacs.2022.100368

**Published:** 2022-05-14

**Authors:** Hiroyuki Fujii, Iori Terabayashi, Kazumichi Kobayashi, Masao Watanabe

**Affiliations:** Division of Mechanical and Space Engineering, Faculty of Engineering, Hokkaido University, Kita 13 Nishi 8, Kita-ku, Sapporo, Hokkaido 060-8628, Japan

**Keywords:** Modeling photoacoustic pressure generation, Grüneisen parameter, Light scattering properties, Hard-sphere interaction between colloidal particles, Multi-scale approach

## Abstract

Further development of quantitative photoacoustic tomography requires understanding the photoacoustic pressure generation by modeling the generation process. This study modeled the initial photoacoustic pressure in colloidal suspensions, used as tissue phantoms, at different volume fractions on a multi-scale approach. We modeled the thermodynamic and light scattering properties on a microscopic scale with/without treating the hard-sphere interaction between colloidal particles. Meanwhile, we did the light energy density on a macroscopic scale. We showed that the hard-sphere interaction significantly influences the initial pressure and related quantities at a high volume fraction except for the thermodynamic properties. We also showed the initial pressure at the absorber inside the medium logarithmically decreases with increasing the volume fractions. This result is mainly due to the decay of the light energy density with light scattering. Our numerical results suggest that modeling light scattering and propagation is crucial over modeling thermal expansion.

## Highlights

• Modeling the initial photoacoustic pressure based on a multi-scale approach.
• Modeling the thermodynamic and light scattering properties on the microscale.
• Strong influences of the hard-sphere interaction on the initial pressure.
• Light scattering mainly contributes to the pressure over the thermal expansion.

## Graphical abstract

**Figure:**
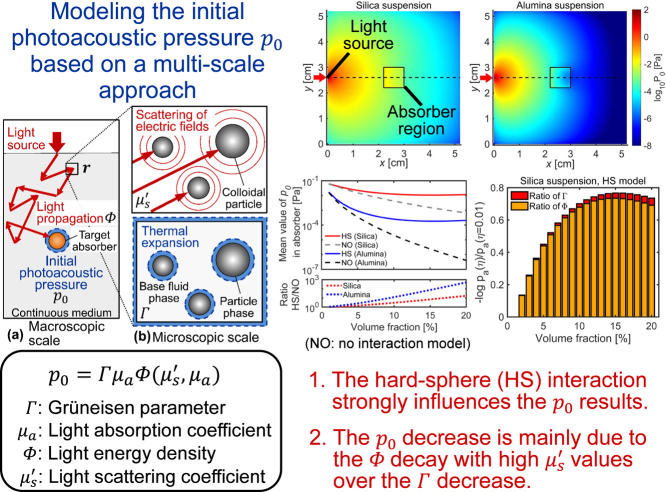

## 1 Introduction

Photoacoustic imaging (PAI), also called optoacoustic imaging, enables evaluating chemical components (e.g., hemoglobin concentration) for biological tissues and foods better than pure optical imaging [1], [2], [3], [4], [5]. Although PAI has been rapidly developed, quantitative photoacoustic tomography (QPAT) is still growing, which aims the quantitative evaluation deeply inside the media. The QPAT development requires understanding photoacoustic pressure generation, that is, the initial photoacoustic pressure, by modeling the generation process: laser irradiation, light propagation, light absorption, conversion from optical energy to thermal energy, thermal expansion, etc. The initial pressure includes the information of the light absorption coefficient, which directly correlates with the chemical components. The understanding of the initial pressure provides valuable knowledge for QPAT, e.g., a way of evaluating the absorption coefficient by separating from the other contributions, such as thermal expansion. The pressure generation involves a multi-scale process from a microscopic scale (micrometer-scale) to a macroscopic scale (centimeter-scale). The light energy propagates on the macroscopic scale in a medium through multiple light scattering and absorption events. On the macroscopic scale, the medium is considered to be continuous. Meanwhile, thermal expansion, light scattering, and light absorption occur on the microscopic scale in an infinitesimal volume of the continuous medium. In the near-infrared wavelength used in PAI, the light scattering is dominant over the light absorption. The Grüneisen parameter (GP) characterizes the thermal efficiency for the initial pressure and consists of the specific heat capacity at constant pressure, thermal (or volumetric) expansion coefficient, and adiabatic sound velocity [1]. Various initial pressure models have been developed, such as a combined model with the light propagation model on the macroscopic scale [6], [7], [8], [9], [10]. However, to the best of our knowledge, a multi-scale model has not been developed by combining models of the GP and light scattering properties on the microscopic scale; and light energy density on the macroscopic scale.

A colloidal suspension, such as silica and alumina suspensions, has been used as a liquid tissue phantom for calibration and standardization of optical imaging techniques, including PAI [9], [11], [12], [13], [14], [15], because we can adjust the optical properties of the suspension with high stability. Because the volume fraction (or concentration) is a control parameter for the suspension, understanding the volume fraction dependence of the initial pressure and related properties (e.g., the GP and light scattering properties) is indispensable. Our first objective is to develop the initial pressure model for colloidal suspensions (aqueous silica and alumina suspensions) at different volume fractions up to 20% based on the multi-scale approach. Mainly, we focus on the GP among the related properties.

Determination of the GP is still challenging because the GP depends on temperature, volume fraction (or concentration), kinds of materials, etc., in a complicated way. Moreover, the direct measurement of the GP is not straightforward, and we mostly need to measure the related thermodynamic properties separately. Previous studies have extensively discussed the temperature dependence of the GP for biological tissue volumes and tissue phantoms in biomedical optics [12], [16], [17], while they have less discussed the volume fraction dependence. Laufer et al. have examined the experimental results of the GP for aqueous suspensions of copper and nickel chloride using a linear-concentration-dependent model [18]. They have shown an increase in the GP values with increasing the concentration. Meanwhile, Yao et al. have discussed the decreasing trend of the GP values for the lipid–water mixture [16]. These results indicate the complicated dependence of the GP on the volume fraction.

In thermal and fluid engineering, extensive studies have examined the thermodynamic properties for colloidal suspensions by a weight-based (WB) model [19] and the Urick model [20]. The WB and Urick models rely on the mixing rule for the dispersed particle phase and base fluid phase. The numerical results using the two models agree with experimental results at different volume fractions up to roughly 20% for the specific heat capacity [19] and 5% for the sound velocity [21]. Because the two models consider no interaction between colloidal particles, the agreements indicate less influences of the particle interaction on the thermodynamic properties at the volume fraction ranges. At a higher volume fraction and for other properties, meanwhile, the influence is still unclear. In applied physics, the Carnahan–Starling (CS) model has been widely used to describe the thermodynamic properties for a single-component liquid, such as liquid metals [22], [23], [24]. The numerical results using the CS model agree with the experimental data by treating the hard-sphere interaction between the particles. However, the application of the CS model to colloidal suspensions is not straightforward because this model does not consider mixing the two phases. We develop a model for the GP and related thermodynamic properties in colloidal suspensions by combining the WB or Urick model with the CS model. The developed models simply treat the hard-sphere interaction and mixing rule.

In diffuse optics, many researches have discussed the light scattering properties for colloidal suspensions using the dependent and independent scattering theories (DST and IST) [25], [26], [27], [28], [29]. The DST well describes measurement data at different volume fractions up to approximately 20%, while the applicability of the IST limits to a few percent [30], [31], [32], [33], [34]. Unlike the IST, the DST treats the interference of the electric fields scattered by colloidal particles [35], where the interference depends on the two-particle correlation. Most studies have calculated the correlation by the Percus–Yevick hard-sphere model, similar to the CS model. This fact means the hard-sphere interaction strongly influences the light scattering properties over other particle interactions, such as the van der Waals interaction at the volume fraction range. Moreover, our previous numerical study has shown the hard-sphere interaction influences the light energy density [36], [37]. However, the effect of the hard-sphere interaction on the initial photoacoustic pressure is unclear. Our second objective is to clarify the influence using the developed initial pressure models with/without treating the hard-sphere interaction.

In the next section, we explain mainly the models of the GP and related thermodynamic properties and briefly explain the models of light scattering properties and light energy density. Section 3 provides the numerical results for the models and discussion. Section 4 concludes the remarks.

## 2 Modeling initial photoacoustic pressure based on a multi-scale approach

**Fig. 1:**
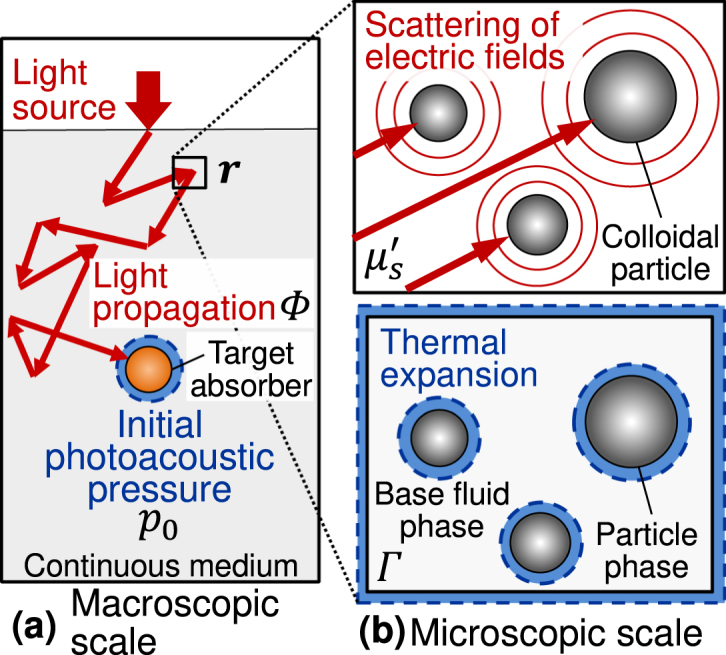
Schematic of the initial photoacoustic pressure model for a colloidal suspension based on the multi-scale approach: (a) macroscopic and (b) microscopic scales. In the figure (b)’s top, the red arrow represents the incident direction of the electric fields and red spherical waves the scattered fields from colloidal particles. In the figure (b)’s bottom, blue regions represent the small volume changes by thermal expansion.

The initial photoacoustic pressure $p_{0}\left(r\right)$ at the position r is given as

(1) $$p_{0}\left(r;\eta \right)=\Gamma \left(\eta \right)\mu_{a}\left(r\right)\Phi \left(r;\eta ,\mu_{s}^{\prime },\mu_{a}\right),$$

where Γ is the Grüneisen parameter (GP), $\mu_{a}\left(r\right)$ the light absorption coefficient, and Φ(r) the light energy density (or fluence rate). We considered colloidal suspensions at different volume fractions η[−] of the particles from 0.01 to 0.20. The water content of biological tissues (e.g., the human lung and human brain) is approximately 0.8 [38]. Hence, the volume fraction range up to 0.2 in the current study seems reasonable. Φ(r) depends on the light scattering properties (e.g., the reduced scattering coefficient $\mu_{s}^{\prime }$) and $\mu_{a}$. Fig. 1 shows the schematic of the $p_{0}$ model based on the multi-scale approach. The initial pressure is generated locally from each infinitesimal volume in the continuous medium, while the light energy propagates on the macroscopic scale (Fig. 1(a)). We modeled the thermodynamic and light scattering properties on the microscopic scale (Fig. 1(b)), where the discrete colloidal particles are suspended in a base fluid (water in this study) with/without treating the hard-sphere interaction between the particles. The particles are correlated with each other by the hard-sphere model, while no correlation for the no interaction model [24], [35]. Table 1 lists the models for the three quantities (Γ, $\mu_{s}^{\prime }$, Φ(r)) to calculate $p_{0}\left(r\right)$. We considered a single optical wavelength of 600 nm. We set the $\mu_{a}$-values of 0.5 ${\mathrm{cm}}^{-1}$ for a single absorber and 0.05 ${\mathrm{cm}}^{-1}$ for the other region of the continuous medium, independently of the volume fraction. Meanwhile, we considered homogeneous distributions of the thermodynamic and light scattering properties at each volume fraction.

**Table 1:** Modeling the volume fraction dependence of the thermodynamic and light scattering properties on the microscopic scale and light energy density on the macroscopic scale. The microscopic models treat no interaction between particles or hard-sphere interaction by the Carnahan–Starling (CS) and Percus–Yevick (PY) models.

Physical quantities	No (NO) interaction	Hard-sphere (HS) interaction
Thermodynamic properties (microscopic scale)	WB model, Urick model	WCS model, UCS model

Light scattering properties (microscopic scale)	Independent scattering theory (IST)	Dependent scattering theory (DST) with PY model

Light energy density (macroscopic scale)	Photon diffusion equation (PDE)	

### 2.1 Modeling thermodynamic properties

The GP, Γ[−], is defined by $\beta V^{2}/C=\beta /\left(\rho \kappa C\right)$, where $\beta \;\left[K^{-1}\right]$ is the thermal (or volumetric) expansion coefficient, V[m/s] is the adiabatic sound velocity, $\rho \;\left[\mathrm{kg}/m^{3}\right]$ is the mass density, $\kappa \;\left[{\mathrm{Pa}}^{-1}\right]$ is the isothermal compressibility, and C[J/kgK] is the specific heat capacity at constant pressure. We modeled the η-dependence of the above related thermodynamic properties for Γ-calculations.

#### 2.1.1 Weight-based (WB) and Urick models (no interaction models)

The WB model provides the formulations of ρ, C, and β based on the mixing rule for the dispersed phase of colloidal particles and base fluid phase (Fig. 1(b)’s bottom), where the thermal equilibrium between the two phases is assumed [19]. We denote the thermodynamic coefficients for the particle phase and water (base fluid) phase by subscripts p and w, respectively (e.g., the mass densities for the two phases $\rho_{p}$ and $\rho_{w}$). The coefficients are dependent on temperature T but independent of η. The mass density ρ for a colloidal suspension at a volume fraction η is given as

(2) $$\rho \left(\eta \right)=\eta \rho_{p}+\left(1-\eta \right)\rho_{w}.$$

Formulations of C and β using the WB model are given as

(3) $$C_{WB}\left(\eta \right)=\rho^{-1}\left(\eta \right)\left[\eta \rho_{p}C_{p}+\left(1-\eta \right)\rho_{w}C_{w}\right],$$

(4) $$\beta_{WB}\left(\eta \right)=\rho^{-1}\left(\eta \right)\left[\eta \rho_{p}\beta_{p}+\left(1-\eta \right)\rho_{w}\beta_{w}\right],$$

where $C_{p}$, $\beta_{p}$, $C_{w}$, and $\beta_{w}$ are the values of C and β for each phases.

The Urick model is based on the linear mixing rule for the two phases and valid under the condition (so-called long-wavelength requirement) that the acoustic wavelength is much longer than the particle size [39]. The Urick model provides a V-formulation for the colloidal suspension [20]

(5) $$V_{U}\left(\eta \right)={\left[\rho \left(\eta \right)\kappa_{U}\left(\eta \right)\right]}^{-1/2},$$

(6) $$\kappa_{U}\left(\eta \right)=\eta \kappa_{p}+\left(1-\eta \right)\kappa_{w}.$$

Here, $\kappa_{p}$ and $\kappa_{w}$ are the κ-values for each phases. Using the WB and Urick models, we calculate the η-dependence of Γ as

(7) $$\Gamma_{NO}=\frac{\beta_{WB}V_{U}^{2}}{C_{WB}}=\frac{\beta_{WB}}{\rho \kappa_{U}C_{WB}}.$$

The WB and Urick models do not treat the interaction between the particles.

#### 2.1.2 WCS and UCS models (hard-sphere models), combined with the Carnahan–Starling (CS) model

In this study, we developed models of the thermodynamic properties to treat the hard-sphere interaction and the mixing rule for the two phases by combining the WB or Urick models with the CS model. We refer the developed models to the WCS and UCS models, respectively.

The CS model treats the hard-sphere interaction for the thermodynamic properties in a single-component liquid and provides the expression for the equation of state [22]. Using the equation of state, the original formulation of β using the CS model is given as [23]

(8) $$\beta_{CS,original}\left(\eta \right)=\frac{1}{T}\frac{1-2\eta^{3}+\eta^{4}}{1+4\eta +4\eta^{2}-4\eta^{3}+\eta^{4}}.$$

In Eq. (8), the β-value at η=0 is given as 1/T, equivalent to the value for the ideal gas (no interaction system). The limit is appropriate for a single-component liquid, but it is not for a colloidal suspension, where the β-value at η=0 should be the value for a base fluid ($\beta_{w}$ in our study). The CS model does not treat the mixing rule for the two phases. We proposed a β-formulation for a colloidal suspension using the WCS model

(9) $$\beta_{WCS}\left(\eta \right)=\rho^{-1}\left(\eta \right)\left[\eta \rho_{p}\beta_{CS}\left(\eta \right)+\left(1-\eta \right)\rho_{w}\beta_{w}\right],$$

$$\beta_{CS}\left(\eta \right)=\beta_{p}\frac{1-2\eta^{3}+\eta^{4}}{1+4\eta +4\eta^{2}-4\eta^{3}+\eta^{4}}.$$

The $\beta_{CS}$-formulation is obtained by replacing the factor 1/T in Eq. (8) with the coefficient $\beta_{p}$, and $\beta_{CS}\left(\eta \right)$ treats the hard-sphere interaction. The coefficient $\beta_{p}$ is the same as which appears in the WB model (Eq. (4)) and is independent of η. Eq. (9) satisfies the limit condition at η=0; $\beta_{WCS}\left(0\right)=\beta_{w}$. In the WCS model, the formulation of the mass density (Eq. (2)) is not modified because of mass conservation.

In the same manner to Eq. (9), we proposed formulations of C using the WCS model and κ using the UCS model, respectively

(10) $$C_{WCS}\left(\eta \right)=\rho^{-1}\left(\eta \right)\left[\eta \rho_{p}C_{CS}\left(\eta \right)+\left(1-\eta \right)\rho_{w}C_{w}\right],$$

$$C_{CS}\left(\eta \right)=C_{p}\frac{{\left(1-2\eta^{3}+\eta^{4}\right)}^{2}}{\left(1+4\eta +4\eta^{2}-4\eta^{3}+\eta^{4}\right){\left(1-\eta \right)}^{4}},$$

(11) $$\kappa_{UCS}\left(\eta \right)=\eta \kappa_{CS}\left(\eta \right)+\left(1-\eta \right)\kappa_{w},$$

$$\kappa_{CS}\left(\eta \right)=\kappa_{p}\frac{{\left(1-\eta \right)}^{4}}{1+4\eta +4\eta^{2}-4\eta^{3}+\eta^{4}}.$$

We obtained the η-dependence of $C_{CS}\left(\eta \right)$ and $\kappa_{CS}\left(\eta \right)$ from the original expressions for the CS model. The original expressions of the CS model for the C and κ are given as

(12) $$C_{CS,original}\left(\eta \right)=\frac{3}{2}k_{B}+k_{B}\frac{{\left(1-2\eta^{3}+\eta^{4}\right)}^{2}}{\left(1+4\eta +4\eta^{2}-4\eta^{3}+\eta^{4}\right){\left(1-\eta \right)}^{4}},$$

(13) $$\kappa_{CS,original}\left(\eta \right)=\frac{1}{n_{0}k_{B}T}\frac{{\left(1-\eta \right)}^{4}}{1+4\eta +4\eta^{2}-4\eta^{3}+\eta^{4}},$$

where $k_{B}$ is the Boltzmann constant and $n_{0}$ is the number density for a system. The Ref. [23] provides the formulation of $\kappa_{CS,original}\left(\eta \right)$, while we calculate the formulation of $C_{CS,original}\left(\eta \right)$ in the same way as the Ref. [23]. The coefficients $C_{p}$ and $\kappa_{p}$ are the same as those in Eqs. (3), (6). Using the WCS and UCS models (hard-sphere models), we calculate the η-dependence of Γ as

(14) $$\Gamma_{HS}=\frac{\beta_{WCS}V_{UCS}^{2}}{C_{WCS}}=\frac{\beta_{WCS}}{\rho \kappa_{UCS}C_{WCS}}.$$

Here, the sound velocity $V_{UCS}$ for the UCS model is given as

(15) $$V_{UCS}\left(\eta \right)={\left[\rho \left(\eta \right)\kappa_{UCS}\left(\eta \right)\right]}^{-1/2}.$$

#### 2.1.3 Numerical conditions of the models for the thermodynamic properties

We focus on aqueous silica and alumina suspensions at different volume fractions up to basically 20%. We used the thermodynamic coefficients for the two phases (e.g., $C_{p}$ and $C_{w}$) from references. Table 2 lists values of the coefficients for silica particles (mean diameter range of 20–80 nm), alumina particles (diameter range of 33–50 nm), and water. Slavova et al. [40] provided the $\rho_{p}$-value of 2196 $\mathrm{kg}/m^{3}$ for several kinds of silica suspensions (Ludox SM, HS-30, TM-50, Sigma-Aldrich), independently of the particle diameter in the 3–11 nm range. Horváth-Szabó and Høiland [41] used the $\rho_{p}$-value of 2182 $\mathrm{kg}/m^{3}$ for the silica particles with the mean diameter of 80 nm. Based on the two references, we determined the $\rho_{p}$-value as 2190 $\mathrm{kg}/m^{3}$ at the diameter of 40 nm by linear interpolation. Horváth-Szabó and Høiland [41] also determined the $\kappa_{p}$-value for silica particles at the diameter of approximately 80–150 nm, independent of the diameter. Mahrholz et al. [42] reported the $\beta_{p}$-value range of 0.5$\;\times \;10^{-6}$-0.9$\;\times \;10^{-6}\;K^{-1}$ for silica particles in the 8–50 nm diameter range. Based on the $\beta_{p}$-value range, we determined the $\beta_{p}$-value as 0.8$\;\times \;10^{-6}\;K^{-1}$ at the diameter of 40 nm by linear interpolation. It is worth noting that the $\beta_{p}$-value for silica particles is smaller than those for other particles (e.g., alumina) because the amorphous silica particle has a network structure [43]. Although the values of the coefficients for alumina particles have been determined in the 33–50 nm diameter range, we assumed their size dependence is small. All the values listed in Table 2 have been evaluated in the T-range of 25–30 °C. Although the coefficient values depend on T, the dependence is small in the short T-range.

**Table 2:** Thermodynamic coefficients of silica particles, alumina particles, and water at room temperature; the mass density ρ, specific heat capacity at constant pressure C, isothermal compressibility κ, and thermal expansion coefficient β. The subscripts of p and w represent the values for particles and water, respectively. As remarks to the values for the particles, a mean diameter is denoted.

Thermodynamic coefficients	Materials	Values	Remarks
$\rho_{p}$ [kg/m${}^{3}$]	Silica	2190	Ref. [40], [41], 40 nm
	Alumina	3600	Ref. [44], 45 nm
$\rho_{w}$ [kg/m${}^{3}$]	Water	997	–

$C_{p}$ [J/kg K]	Silica	745	Ref. [45], 20 nm
	Alumina	775	Ref. [21], 50 nm
$C_{w}$ [J/kg K]	Water	4180	Ref. [45]

$\kappa_{p}$ [Pa${}^{-1}$]	Silica	$2.80\;\times \;10^{-11}$	Ref. [41], 80 nm
	Alumina	$3.94\;\times \;10^{-12}$	Ref. [21], 50 nm
$\kappa_{w}$ [Pa${}^{-1}$]	Water	4.48 × 10${}^{-10}$	Ref. [41]

$\beta_{p}$ [K${}^{-1}$]	Silica	0.80 × 10${}^{-6}$	Ref. [42], 40 nm
	Alumina	0.85 × 10${}^{-5}$	Ref. [46], 33 nm
$\beta_{w}$ [K${}^{-1}$]	Water	2.37 × 10${}^{-4}$	Ref. [19]

### 2.2 Modeling light energy density

For modeling the light energy density Φ(r), we employed the photon diffusion equation (PDE), which is the approximation to the radiative transfer equation (RTE) [47], [48]. The RTE and PDE describe the light energy propagation on the macroscopic scale (Fig. 1(a)). We preliminary confirmed that the PDE results agree with the RTE results at most spatial regions, especially inside the medium for the two suspensions. For the alumina suspension, please see our previous study [49]. We numerically calculated the PDE using the finite difference method with the spatial grid size of 0.04 cm for a cubic heterogeneous medium with a size of 5.2 cm. The medium consists of a single cubic absorber of $\mu_{a}=0.5\;{\mathrm{cm}}^{-1}$ with a size of 0.8 cm on the center and a homogeneous background ($\mu_{a}=0.05\;{\mathrm{cm}}^{-1}$). The PDE calculation requires a value of the reduced scattering coefficient $\mu_{s}^{\prime }$ as the light scattering properties. We adopted the numerical results of $\mu_{s}^{\prime }$ using the independent and dependent scattering theories (IST and DST).

### 2.3 Modeling light scattering properties

The IST and DST provide the formulations of the scattering properties for colloidal suspensions at different volume fractions on the microscopic scale (Fig. 1(b)’s top). The IST and DST also refer to the zeroth-order and first-order scattering theories because the two theories are the zeroth-order and first-order solutions of the Foldy-Lax equation [35], which is the multiple scattering expansion of the Maxwell equations [50], [51]. The IST treats no interaction of the electric fields scattered by the particles, while the DST treats the interference of the fields in the far-field. The static structure factor describes the contribution of the interference and represents the two-particle correlation. We calculated the factor by the Percus–Yevick (PY) model [52], [53], which treats the hard-sphere interaction between the particles. The calculations of the IST and DST need values of the refractive indices for particles and water (base fluid), $n_{p}$ and $n_{w}$, and the particle size distributions. We referred to the $n_{p}$-values as 1.470 for silica, 1.768 for alumina, and the $n_{w}$-value as 1.332 for water [54], [55] with the optical wavelength of 600 nm. We adopted the particle size distributions of silica measured by Horváth-Szabó and Høiland (mean value of 83 nm) [41] and alumina by Mahmoud et al. [21] (mean value of 55 nm), respectively. Please see the references, including our previous work, for the explicit formulations of the IST and DST and numerical conditions [27], [36].

## 3 Results and discussion

### 3.1 Thermodynamic properties, including the GP

In this subsection, we calculated the η-dependence of the thermodynamic properties using the four models (WB, Urick, WBCS, UCS models). We tested the validity of the models by comparing the numerical results with the experimental results of C(η) and V(η) for aqueous silica and alumina suspensions. We numerically investigated the influences of the hard-sphere interaction on the thermodynamic properties.

#### 3.1.1 Specific heat capacity and thermal expansion coefficient

**Fig. 2:**
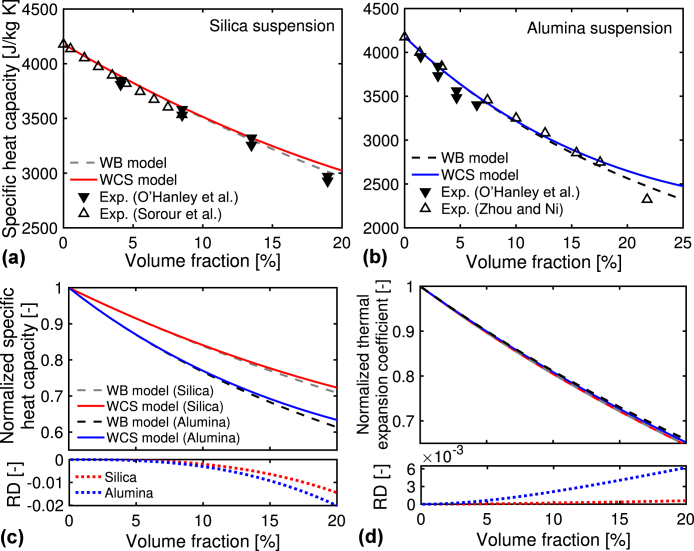
(a, b) Specific heat capacity at constant pressure C for the silica and alumina suspensions at different volume fractions; numerical results for the WB and WCS models (Eqs. (3), (10)), and experimental results. We used the measurement data of (a) silica suspensions by O’Hanley et al. [56] and Sorour et al. [57]; and (b) alumina suspensions by O’Hanley et al. [56] and by Zhou and Ni [44]. (c) Normalized C-results by the water value $C_{w}$ using the two models for the two suspensions. (d) Normalized thermal expansion coefficient by the water value $\beta_{w}$ using the two models (Eqs. (4), (9)). Figures (c) and (d)’s bottom plots the relative differences (RD) in the normalized results between the two models.

Fig. 2(a) and (b) compare the numerical results of specific heat capacity C(η) using the WB and WCS models (Eqs. (3), (10)) with the experimental results. Here, we used the measurement data of silica suspensions with the mean particle diameter of 32 nm (Ludox TMA) at 35 °C by O’Hanley et al. [56] and the diameter of 8 nm (Ludux SM-30) at 30 °C by Sorour et al.; and alumina suspensions with the diameter of 50 nm (AL20DW) at 35 °C by O’Hanley et al. [56] and the diameter of 45 nm at 33 °C by Zhou and Ni [44]. The numerical results for the two models agree with the experimental results, suggesting the validations of the two models. Despite the differences in the particle diameters and temperatures between the models and measurements, the agreement indicates that the differences do not significantly influence the C-results.

Fig. 2(c) shows the normalized C-results by the water value $C_{w}$ using the two models for the two suspensions. As the volume fraction increases to 20%, the normalized C-values decrease to approximately 0.7 in silica suspension and 0.6 in alumina suspension, respectively. The decrease in the C-values means the suspension takes less energy for a temperature rise as particles are added to the suspension. The decrease mainly comes from mixing the particle phase and base fluid phase. At the bottom of Fig. 2(c), we calculated the relative difference (RD) in the normalized C-results between the two models. We define the RD as NR(WB)−NR(WCS) with the normalized results for the WB and WCS models, NR(WB) and NR(WCS), respectively. The RD becomes negatively larger with increasing the volume fraction, meaning the enhancement of the hard-sphere interaction. However, the RD-value is much smaller than the decrease in the C-results with increasing the volume fraction. This fact means the hard-sphere interaction less contributes to the C-results than the mixing for the two phases.

Fig. 2(d) shows the numerical results of the thermal expansion coefficient β(η) using the WB and WCS models (Eqs. (4), (9)), where the water value $\beta_{w}$ normalizes the results. As the volume fraction increases to 20%, the β-values for both suspensions decrease to approximately 0.65. Such decreasing trend has been observed in other systems, such as silica-poly(vinyl acetate) mixture [43]. As shown in the figure’s bottom, the RD-values between the two models are very small for the two suspensions, meaning minor influences of the hard-sphere interaction to the β-results. This result is because the $\beta_{p}$-value is quite smaller than the $\beta_{w}$-value (one or two digits smaller, as listed in Table 2).

#### 3.1.2 Sound velocity, isothermal compressibility, and Grüneisen parameter (GP)

**Fig. 3:**
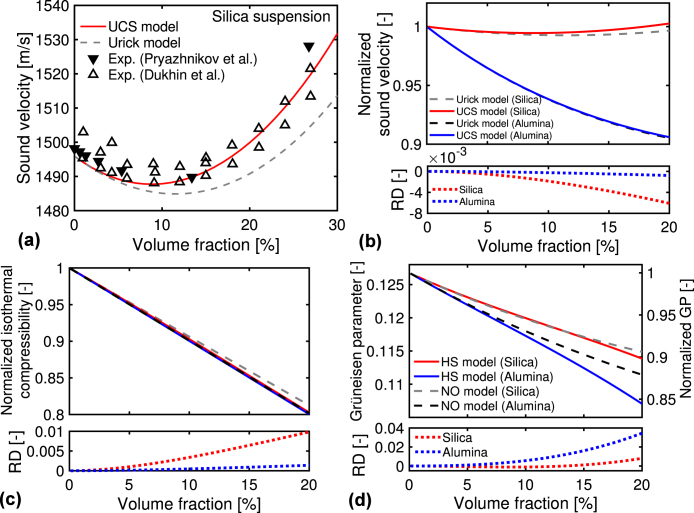
(a) Sound velocity V for the silica suspension at different volume fractions; numerical results for the Urick and UCS models (Eqs. (5), (15)); and experimental results by Pryazhnikov and Minakov [58] and by Dukhin et al. [59]. (b) Normalized V-results by the water value ${\left(\rho_{w}\kappa_{w}\right)}^{-1/2}$ for the silica and alumina suspensions using the two models. (c) Isothermal compressibility κ normalized by the water value $\kappa_{w}$ using the Urick and UCS models (Eqs. (6), (11)). (d) Grüneisen parameter (GP), Γ, using the no interaction (NO) and hard-sphere (HS) models (Eqs. (7), (14)). On the right axis, the normalized Γ-results by the water value of 0.127 are plotted.

Fig. 3(a) compares the numerical results of sound velocity V(η) using the Urick and UCS models (Eqs. (5), (15)) with the experimental results for the silica suspension with the mean particle diameter of 22 nm (Ludox TM-50) at 25 °C by Pryazhnikov and Minakov [58] and with the diameter of 30 nm (Ludox TM) by Dukhin et al. [59]. The experimental studies consider the frequency range of 3–100 MHz and V-range of 1480–1530 m/s, resulting in the acoustic wavelength of 10^−4^ m. Hence, the experimental studies satisfy the long-wavelength requirement of the Urick model. The numerical V-results for the two models, especially the UCS model, agree with the experimental results, suggesting the validity of the two models. The agreement suggests that the hard-sphere interaction is dominant over other particle interactions, such as the van der Waals interaction. It is noted that the volume fraction (or concentration) dependence of V varies at different systems, e.g., the decrease in the V-values with increasing the concentrations for liquid emulsions, while the increasing trend for aqueous gels [18].

In Fig. 3(b), we plot the V-results normalized by the water value ${\left(\rho_{w}\kappa_{w}\right)}^{-1/2}$ using the two models. As the volume fraction increases, the V-results for the alumina suspension monotonically reduce from the water value. Meanwhile, the V-results for the silica suspension slightly decrease in the volume fraction range of 1%–10% and increase in the 10%–20% range. Fig. 3(c) shows the numerical results of isothermal compressibility κ(η) normalized by the water value $\kappa_{w}$. The κ-difference between the two suspensions is smaller than the κ-change to the volume fraction. Hence, the V-difference between the two suspensions mainly comes from the $\rho_{p}$-difference rather than the $\kappa_{p}$-difference. As shown in Fig. 3(b) and (c)’s bottom, the RD-values of V and κ between the Urick and UCS models (NR(Urick)−NR(UCS)) are much smaller than the changes in V and κ to the volume fractions. This fact indicates the hard-sphere interaction less contributes to the results of V and κ than the mixing for the two phases, similar to the results of C and β.

Fig. 3(d) shows the numerical results of the GP, Γ(η), using the two models: the no interaction (NO) model (Eq. (7)) and hard-sphere (HS) model (Eq. (14)). As the volume fraction increases to 20%, the Γ-values decrease to around 0.107 for the HS model and 0.115 for the NO model, respectively (see the left axis). The decreasing behavior for the Γ-values has been reported in an experimental study for lipid–water mixture [16] and first-principles molecular dynamics simulations of silica liquid [60]. Meanwhile, the increasing Γ behavior has been reported for aqueous suspensions of copper and nickel chloride [18], [61]. This difference in the Γ behaviors is probably caused by the difference in the thermodynamic coefficients for the materials. We plot the normalized Γ-values on the right axis by the water value $\beta_{w}/\left(\rho_{w}\kappa_{w}C_{w}\right)=0.127$. With increasing the volume fraction, the normalized values decrease by approximately 0.15 for the HS model and 0.10 for the NO model, respectively. The decreasing Γ-values are smaller than the changes in the thermodynamic properties, such as C and β (See Fig. 2(c) and (d)). This result is because the changes in the denominator and numerator of $\Gamma =\beta V^{2}/C$ are canceled out of each other. As shown in the figure’s bottom, the RD-values of Γ between the two models (NR(NO)−NR(HS)) increases to 0.04. The increase in the RD-values is a quarter of the decrease in Γ-values with increasing the volume fractions. This result is similar to the results for the related thermodynamic properties, indicating the minor contribution of the hard-sphere interaction over the contribution of mixing for the two phases.

### 3.2 Light scattering properties and light energy density

**Fig. 4:**
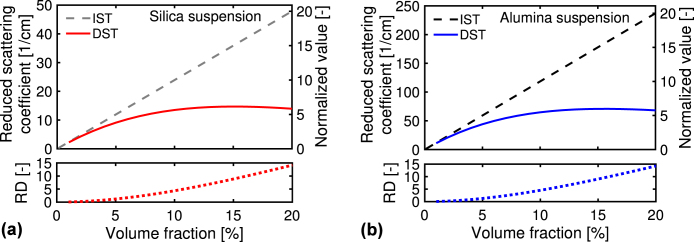
Reduced scattering coefficients at different volume fractions for silica and alumina suspensions using the IST (no interaction) and DST (hard-sphere interaction). On the right axis, the normalized values by the result for the IST at the volume fraction of 1% are plotted.

Fig. 4(a) and (b) show the reduced scattering coefficient, $\mu_{s}^{\prime }\left(\eta \right)$, using the IST (no interaction) and DST (hard-sphere interaction) for the two suspensions. The $\mu_{s}^{\prime }$-values for the alumina suspension are approximately five times larger than those for the silica suspension (see the left axis), mainly because the refractive index for the alumina particles is larger than that for the silica particles. As the volume fraction increases, the $\mu_{s}^{\prime }$-value becomes more significant, meaning the enhancement of the light scattering. In the figure’s bottom, we plot the RD-values, defined as NR(IST)−NR(DST). NR(IST) and NR(DST) represent the results using the IST and DST normalized by the IST result at the volume fraction of 1%, respectively. Although the $\mu_{s}^{\prime }$-values differ from both suspensions, the RD-values are almost the same and increase to around 15 with increasing the volume fraction. This result means the contribution of the hard-sphere interaction depends less on a kind of material. We plot the normalized $\mu_{s}^{\prime }$-values on the right axis by the result using the IST at the volume fraction of 1%. For the two suspensions, the normalized values for the DST increase to around 5 with increasing the volume fraction. The RD-value of $\mu_{s}^{\prime }$ is about three times larger than the change in the normalized value for the DST to the volume fraction. Meanwhile, as shown in Fig. 3(d), the RD-value of Γ is a quarter of the change in the normalized value. These results suggest the hard-sphere interaction strongly influences light scattering properties compared to the thermodynamic properties.

**Fig. 5:**
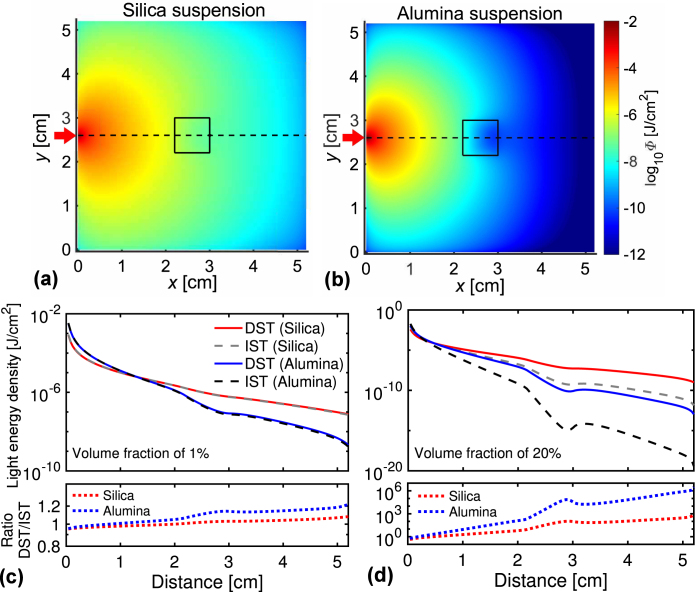
(a, b) Spatial distributions of the light energy density Φ at a plane of z=2.6 cm using the PDE with the DST for the two suspensions at the volume fraction of 15%. A red arrow denotes the light source incident on (0.0 cm, 2.6 cm, 2.6 cm); black square a boundary of the single absorber with a size of 0.8 cm. (c, d) The Φ-calculations from the PDE with the DST or IST for the different distances between the source position and the calculating points on the line of (x, 2.6, 2.6) denoted by the black dashed line in the figures (a) and (b). The bottom figures plot the ratio of Φ-results using the DST with those using the IST.

**Fig. 6:**
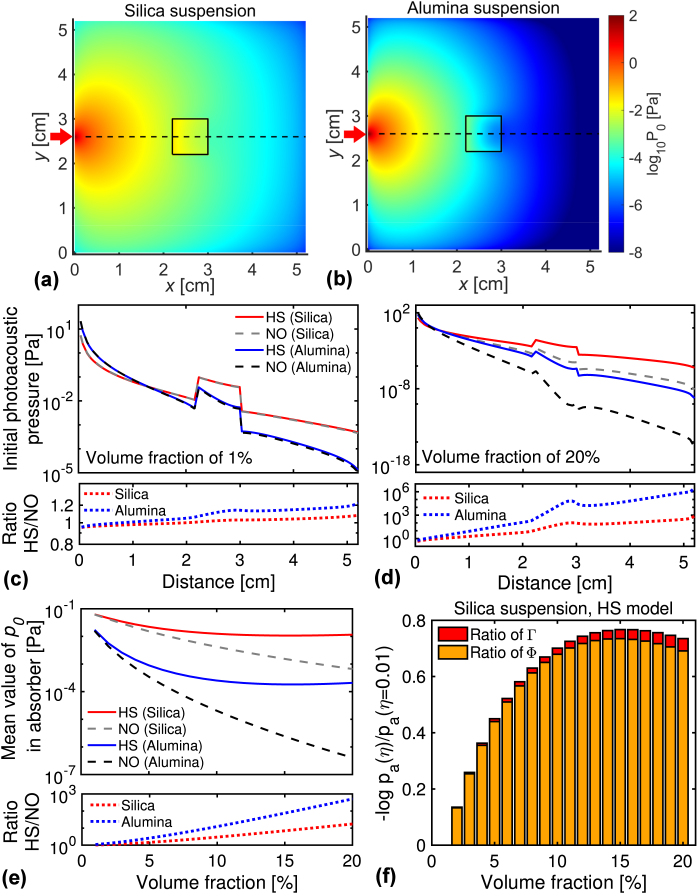
(a, b) Spatial distributions of the initial photoacoustic pressure $p_{0}$ at the plane of z=2.6 cm using the hard-sphere (HS) model (Eq. (16)) for the two suspensions at the volume fraction of 15%. Other details are the same as Fig. 5(a) and (b). (c, d) The $p_{0}$-calculations using the HS model and no interaction (NO) model (Eq. (17)) at the different distances. The bottom figures plot the ratio of $p_{0}$-results using the HS model with those using the NO model. Other details are the same as Fig. 5(c) and (d). (e) Mean values of $p_{0}$ over the absorber region, $p_{a}$, at different volume fractions. (f) Logarithmic $p_{a}$-decrease, $-\log_{10}p_{a}\left(\eta \right)/p_{a}\left(\eta =0.01\right)$, for silica suspensions with the HS model. The $p_{a}$-decrease is decomposed into the ratios of Γ and Φ.

Fig. 5(a) and (b) show the spatial distributions of the light energy density Φ(r;η) at the xy-plane of z=2.6 cm using the PDE with the DST for the two suspensions at the volume fraction 15%. The light source position is (0.0 cm, 2.6 cm, 2.6 cm), denoted by the red arrow. The decay in the Φ-values for the alumina suspension is more significant than that for the silica suspension because of the larger $\mu_{s}^{\prime }$-value for the alumina suspension. We observed the local decay around the absorber (enclosed by the internal square in the figures) for both suspensions. In Fig. 5(c) and (d), we plot the Φ-results with the DST and IST for the different distances between the source position and calculating points on the line of (x, 2.6, 2.6), represented by the black dashed line in Fig. 5(a) and (b). At the volume fraction of 1% (Fig. 5(c)), the Φ-results using the DST almost agree with those using the IST for both the suspensions because the $\mu_{s}^{\prime }$-values are nearly the same between the two theories. At the volume fraction of 20% (Fig. 5(d)), meanwhile, the Φ-results with the DST differ from those with the IST, and the difference becomes significant when the distance becomes large. This result is because of the accumulation of differences in the $\mu_{s}^{\prime }$-values between the DST and IST. In the figure’s bottom, we plot the ratio of the Φ-results using the DST with those using the IST. While the ratios at the volume fraction of 1% are near the unity, the ratios at 20% approach at $10^{6}$, meaning the significant difference in Φ-results between the DST and IST.

### 3.3 Initial photoacoustic pressure

Finally, we examined the initial photoacoustic pressure using the hard-sphere (HS) and no interaction (NO) models,

(16) $$p_{0,HS}\left(r;\eta \right)=\mu_{a}\left(r\right)\Gamma_{HS}\left(\eta \right)\Phi_{DST}\left(r;\eta \right),$$

(17) $$p_{0,NO}\left(r;\eta \right)=\mu_{a}\left(r\right)\Gamma_{NO}\left(\eta \right)\Phi_{IST}\left(r;\eta \right).$$

$\Phi_{DST}$ and $\Phi_{IST}$ are the light energy densities using the DST and IST, respectively. Fig. 6(a) and (b) show the spatial $p_{0}$-distributions using the HS model at the volume fraction of 15%. We observed the logarithmic decay of $p_{0}$ from the light source position, except the absorber region, similar to the Φ-distributions (Fig. 5(a) and (b)). The absorber region has a higher $p_{0}$-value than the surrounding region because the absorber has a higher $\mu_{a}$-value. Nevertheless, the $p_{0}$-values near the source position are much higher than those in the absorber region. This result means the suppression of the photoacoustic generation caused by the decay of the light energy density inside the medium is dominant over the enhancement by the high $\mu_{a}$-value. In Fig. 6(c) and (d), we compared the $p_{0}$-results using the HS model with those using the NO model at different distances between the source and calculating positions. The differences in $p_{0}$-values between the two models are similar to those in Φ-values between the DST and IST. This similarity indicates that the contribution of light propagation (Φ-value) is dominant over that of thermal expansion (Γ-value).

In Fig. 6(e), we calculated the mean values of $p_{0}$ over the absorber region, $p_{a}$, at different volume fractions. At all the volume fractions, the $p_{a}$-values for the HS model are larger than those for the NO model, meaning the hard-sphere interaction suppresses the $p_{0}$-decrease. The ratio of the $p_{a}$-results using the HS model with those using the NO model (shown in the figure’s bottom) logarithmically increases with increasing the volume fraction. In Fig. 6(f), we examined the contribution of the thermal expansion and light propagation (light scattering) on the initial pressure by the logarithmic $p_{a}$-decrease, defined as

(18) $$-\log_{10}\frac{p_{a}\left(\eta \right)}{p_{a}\left(\eta =0.01\right)}=-\log_{10}\frac{\Gamma \left(\eta \right)}{\Gamma \left(\eta =0.01\right)}-\log_{10}\frac{\Phi_{a}\left(\eta \right)}{\Phi_{a}\left(\eta =0.01\right)},$$

where $\Phi_{a}$ is a mean value of Φ over the absorber region. The $p_{a}$-decrease represents the logarithmic reduction from the $p_{a}$-value at the volume fraction of 1% (η=0.01). Because the $\mu_{a}$-value is constant over the volume fraction, the $p_{a}$-decrease is decomposed into the ratios of Γ and Φ. Fig. 6(f) shows the $p_{a}$-decreases at different volume fractions for silica suspension with the HS model. The $p_{a}$-decrease becomes more significant with increasing the volume fractions. The Φ-ratio accounts for more than 94% of the $p_{a}$-decrease at all the volume fractions, while the Γ-ratio does for a few percent. Although not shown here, we obtained similar results of the $p_{a}$-decrease for the other suspension and model. The result means that the light energy density and light scattering properties significantly contributes to the initial pressure than the thermodynamic properties at a wide volume fraction range.

## 4 Conclusions

We have developed the initial photoacoustic pressure model for the colloidal suspensions (aqueous silica and alumina suspensions) at different volume fractions based on the multi-scale approach. Using the developed models, we have examined the influence of the hard-sphere interaction between colloidal particles on the initial pressure and the related physical quantities. We have shown the hard-sphere interaction significantly influences the initial pressure and the related quantities except for the thermodynamic properties. This result suggests that modeling the thermodynamic properties does not strongly require treating the hard-sphere interaction while modeling the light scattering properties should treat it. We have also shown that the initial pressure at the absorber inside the medium logarithmically decreases with increasing the volume fraction. This result is mainly due to the decay of light energy density with light scattering over the decrease in the thermodynamic properties. This result suggests that modeling the light scattering properties and light energy density is crucial over modeling the thermodynamic properties.

Our developed models have the potential to provide valuable information for the quantitative evaluations of the light absorption coefficient from the initial pressure at a deep region of the medium by separating the other contributions, e.g., the Grüneisen parameter and light scattering properties. Our numerical results imply that the separation of light scattering and propagation contributions from the pressure is more significant than the thermal expansion contribution. This numerical study is challenging for future work on quantitative photoacoustic tomography. Our developed models also have the possibility to examine the photoacoustic pressure propagation on the multi-scale approach by combining with the pressure propagation model (e.g., photoacoustic wave equation). An initial pressure model at a higher volume fraction than 20% is significant in future work. Although the average volume fraction of biological tissue volumes is approximately 20%, the volume fraction of the red cells in the whole blood is around 40% [62]. A higher-order scattering theory could be necessary for the light scattering properties at the higher volume fraction. In future work, modeling the initial pressure and related properties to the other colloidal systems is challenging, e.g., charged colloidal suspensions using the Derjaguin–Landau–Verwey–Overbeek (DLVO) interaction model. DLVO interaction strongly influences the structural properties, such as the static structure factor at a high volume fraction [63], implying the influences on the thermodynamic and light scattering properties.

## CRediT authorship contribution statement

**Hiroyuki Fujii:** Conceptualization, Software, Investigation, Funding acquisition, Writing - original draft. **Iori Terabayashi:** Methodology, Software, Formal analysis, Investigation, Writing - review & editing. **Kazumichi Kobayashi:** Writing - review & editing, Supervision. **Masao Watanabe:** Writing - review & editing, Supervision.

## Declaration of Competing Interest

The authors declare that they have no known competing financial interests or personal relationships that could have appeared to influence the work reported in this paper.
